# What has been learned about COVID‐19 vaccine hesitancy in Pakistan: Insights from a narrative review

**DOI:** 10.1002/hsr2.940

**Published:** 2022-11-21

**Authors:** Zoaib Habib Tharwani, Prince Kumar, Wajeeha Bilal Marfani, Sean Kaisser Shaeen, Alishba Adnan, Parvathy Mohanan, Zarmina Islam, Mohammad Yasir Essar

**Affiliations:** ^1^ Faculty of Medicine, Dow Medical College Dow University of Health Sciences Karachi Pakistan; ^2^ Department of MBBS Karachi Medical and Dental College Karachi city Pakistan; ^3^ Department of General Medicine Medical University Sofia Sofia Bulgaria; ^4^ Kabul University of Medical Sciences Kabul Afghanistan

**Keywords:** COVID‐19, Pakistan, vaccine hesitancy

## Abstract

**Background:**

Vaccine hesitancy is not a new phenomenon to Pakistan. This is evidenced through the slow progress of previous vaccination campaigns and programs against MMR, BCG, and especially polio. This issue continues to persist and is therefore becoming the cause of low COVID‐19 vaccination rates in Pakistan.

**Aim:**

To provide insights about COVID‐19 vaccine hesitancy among Pakistanis, and its potential harm on public health. Moreover, we aim provide recommendations to counter the factors limiting the COVID‐19 vaccination in Pakistan.

**Methodology:**

A Boolean search was conducted to find the literature in MEDLINE‐PubMed, Google Scholar, and Clinicaltrials.gov databases up till March 16, 2022. Specific keywords were used which comprised of “SARS‐CoV‐2,” “COVID‐19,” “vaccine hesitancy,” “vaccine acceptance,” “intention to vaccinate,” and “Pakistan,” with use of “OR” and “AND.” Only free full‐text original studies in English language were used to compare and contrast.

**Results:**

As proven by various studies, COVID‐19 vaccination rates are influenced by multiple factors, including inaccurate beliefs about COVID‐19, hesitancy amongst healthcare workers, uncertainty regarding vaccine's efficacy and fear of side effects. Various conspiracy theories and lower testing rates among others also add up to impose a negative impact on the vaccination rates and public health of Pakistan. This may lead to newer strains of potentially harmful COVID‐19, mental health deterioration, and prolonged lockdowns.

**Conclusion:**

Vaccine hesitancy is a global public health threat, and its impacts are pronounced in Pakistan. This is reflected in the COVID‐19 pandemic; low vaccination rates in Pakistan may lead to future outbreaks of new, potentially harmful, strains of COVID‐19 which can prolong lockdowns in the country and affect mental health of the population. To improve the current situations, it is imperative for the government, educational institutes, and healthcare systems to develop trust and continually use dialogue, communication, and education to address misconceptions to improve COVID‐19 vaccination in Pakistan.

## 1 INTRODUCTION

COVID‐19 is an infectious disease caused by severe acute respiratory syndrome coronavirus‐2 (SARS‐CoV‐2). It spreads via inhalation and contact with infected droplets, with an incubation period of 2–14 days, and is capable of causing mild to extreme infections in people. It first appeared in China, in December 2019, and soon advanced to affect the whole world. As of April 3, 2022, more than 491 million cases and over 6 million deaths have been reported worldwide.^1^ Pakistan's first coronavirus case was reported at the Aga Khan University Hospital in Karachi, on February 26, 2020, which then progressed to affect different locales.^2^ Around 1.5 million cases and 30,369 deaths were reported in Pakistan through April 27, 2022.^3^ Amid the pandemic, different variants of COVID‐19 were identified in Pakistan, including alpha, beta, gamma, delta, epsilon, and omicron.^4^, ^5^, ^6^ Despite significant efforts have and are being made to contain the virus, which is circulating to numerous nations with varying degrees of clinical manifestations like fever, cough, tiredness, and ageusia and/or anosmia being the most common symptoms.^7^, ^8^ COVID‐19 can also lead to several neurological dysfunctions other than gustatory and olfactory dysfunctions which include cognition and memory impairments and cranial nerve V3, IX, X, and XII impairment.^9^ Moreover, long‐term consequences include posttraumatic stress disorder (PTSD), depression, grief, and other psychiatric disorders, which may have a negative influence on the perspective of the population towards the disease and vaccination as a prevention strategy.^10^

As of yet, around 64.5% of the world population has received at least the first dose of COVID‐19 vaccine, with approximately 11.29 billion doses administered worldwide.^11^ Currently there are seven vaccines approved for usage in Pakistan against COVID‐19, including Moderna, Pfizer/BioNTech, Convidicea, Sputnik, Oxford/AstraZeneca, Sinopharm (Beijing), and Sinovac (further information on the vaccines available are provided in Table 1).^20^ A booster shot is also recommended due to the concern that efficacy of the vaccine decreases over time, and may prove to become ineffective against a new strain.

**Table 1:** COVID‐19 vaccines in Pakistan and their characteristics

Name of the vaccine	Country of origin	Type of vaccine	Efficacy (%)	Number of doses	Contraindications	Cost in Pakistan
Sinopharm^12^, ^13^	China	Inactivated whole virus	79	2	Individuals with a history of anaphylaxis to any component of the vaccine Anyone with a body temperature over 38.5°C s	Free
Sinovac‐CoronaVac^12^, ^14^	China	Inactivated whole virus	51	2	Individuals with a history of anaphylaxis to any component of the vaccine Persons with acute PCR‐confirmed COVID‐19 Anyone with a body temperature over 38.5°C	Free
CanSino^12^, ^15^	China	Viral Vector	65.7	1	Patients with active COVID infection Individuals on short‐term immunosuppressive medication should wait for 28 days after the medication ends	Free
Sputnik V^12^, ^16^	Russia	Viral vector	91.6	2	Individuals having fever at the time of coming for vaccination Patients with active COVID‐19 Individuals who had severe post vaccination complication with component I should not receive component II	Paid
AstraZeneca^12^, ^17^	United Kingdom	Viral vector	76	2	People with a history of severe allergic reaction to any component of the vaccine The vaccine is not recommended for persons younger than 18 years of age People who developed clotting disorder with the first dose of AstraZeneca vaccine Those with a history of heparin‐induced thrombocytopenia and thrombosis (HITT or HIT type 2)	Free
Moderna^18^	USA	mRNA	94.1	2	Individuals who developed myocarditis or pericarditis following the first dose of mRNA‐1273 The vaccine should not be administered to persons younger than 12 years of age pending the results of further studies	Paid
Pfizer BioNTech^19^	USA	mRNA	95	2	People with a history of severe allergic reaction to any component of the vaccine	Paid

Amidst the implementation of vaccination in the country, Pakistan is facing an emerging challenge of public hesitancy against COVID‐19 vaccination. Vaccine hesitancy, which is the delay in acceptance or refusal of vaccines despite its availability,^21^ is not an uncommon phenomenon in Pakistan, as proven by the slow progress of the campaigns against polio in previous years, which have however shown success recently.^22^, ^23^ Considering the sensitive mindset among the population regarding vaccination, any negative convictions against COVID‐19 immunizations could affect the progressing endeavors to conclude the widespread and may precipitate the complications and long‐term effects associated with the pandemic as discussed above. COVID‐19 immunization aversion thus remains an overwhelming challenge against the public wellbeing of Pakistan amid these delicate circumstances. Therefore, this narrative review aims to provide an insight into Pakistan's vaccination drive by comparing findings of different studies, highlighting the perspective of Pakistanis towards COVID‐19 vaccination and efforts made by the government in alleviating the pandemic, and its impacts on public health. Moreover, this review also aims to provide recommendations to counter the low vaccination rates and limit the impacts of it on the public health of Pakistan.

## 2 METHODS

A Boolean search was carried out to find the literature in MEDLINE‐PubMed, Google Scholar, and Clinicaltrials.gov databases up till March 16, 2022. We used specific keywords that consisted of “SARS‐CoV‐2,” “COVID‐19,” “vaccine hesitancy,” “vaccine acceptance,” “intention to vaccinate,” and “Pakistan,” with interposition of “OR” and “AND.” Full text of all the related articles in English language was retrieved and exported on an excel sheet. The detailed search strategy used is as follows:

(SARS‐CoV‐2 OR COVID OR COVID‐19) AND (vaccine hesitancy OR COVID vaccine acceptance OR intention to vaccinate) AND (Pakistan OR Pakistanis).

The set inclusion criterion for selected articles was: (a) full articles with free access; (b) original studies, observational and interventional studies, randomized controlled trials (if applicable); (c) papers based on the hesitation of the general population of Islamic Republic of Pakistan to get vaccinated for COVID‐19, and (d) articles in English language. However, all the articles in languages other than English; all the reviews, editorials, commentaries, case reports, case series, and articles with abstracts only; and articles targeting the global population were excluded from this review. A total of 43 studies were screened, out of which 12 were excluded due to irrelevance to the topic. The remaining 31 studies were then screened on the basis of full text and six studies were used for this review.

## 3 DISCUSSION

### 3.1 COVID‐19 vaccination statistics in Pakistan

The first COVID‐19 vaccine in Pakistan was given on February 3, 2021.^24^ As of April 10, 2022, more than 249 million vaccine doses have been administered to the general population, which comes out to approximately 113 doses per 100 people.^24^ The rate is somewhat comparable to neighboring countries such as India which despite administering a much higher number of doses, above 1.8 billion till April 11, 2022, still only touched an average of 134.82 doses per 100 people.^24^ This difference is more pronounced with Iran which has around 147 million vaccine doses given out as of April 9, 2022, which comes to 175.09 per 100 people.^24^ However, although lagging behind compared to these two countries, Pakistan is still fairing considerably better compared to another neighboring country, Afghanistan, which has administered only about 5.9 million doses as of April 13, 2022, which comes to a measly 15.26 doses per 100 people.^24^

There are various types and brands of COVID‐19 vaccinations in Pakistan that have been approved for use by the Drug Regulatory Authority of Pakistan, some of which are free of cost to the general public while others being paid. A summary of the brands of vaccines and their efficacy along with other characteristics have been summarized in Table 1.

Luckily, Pakistan has also vastly increased the number of healthcare facilities that can administer the vaccine as UNICEF and the Ministry of National Health Services, Regulations, and Coordination has set up 2800 vaccination centers across the country with an additional 1200 underway. This is supplemented with mobile units being used with the intent to help provide the same vaccinations in underdeveloped and hard to reach rural areas.^25^

### 3.2 The population's perspective

#### 3.2.1 General perspective about vaccination in Pakistan

Vaccination has always been met with skepticism, if not outright hostility, as medical technology.^26^ As far as Pakistan is concerned, vaccine hesitancy is not an anomaly. Due to a limited range of approved therapeutic options, the creation of an effective vaccine has proven to be quintessential in the prevention and mitigation of severe complications such as frequent hospitalizations, which are often accompanied by in‐hospital super‐infections due to decreased immunity.^27^

Various vaccination campaigns have had limited success in Pakistan and multiples challenges have contributed to the high vaccine hesitancy in the country. The country's uncertainty regarding vaccines in part emerges from its low literacy (62.3%)^28^ and low household income rates (587.069 USD per year).^29^, ^30^ The low literacy rates present with a severe challenge to the vaccination efforts, as the locals do not understand the significance of vaccination nor its many benefits, and many fall prey to the conspiracy theories.^31^ Theories like “Polio vaccines are a western plot” are widespread and have hampered vaccination campaigns causing Pakistan to be among the only two countries to still be endemic for polio, despite the recent improvements.^23^, ^32^, ^33^ Religious beliefs are another barrier as many people refuse vaccinations from fears that it might contain pig flesh, which is forbidden for the country's Muslim majority.^34^ Moreover, religious leaders are highly influential in the country and many advise people against vaccines, either due to lack of awareness themselves or in the pursuit of their own political gains.^32^ Vaccination campaigns against MMR, polio, and BCG have been affected by the many stated challenges and thus child mortality in Pakistan is among the worst in the world. Reports from 2020 indicate that there are 65.2 deaths per 1000 livebirths in Pakistan, while the global average is only 37 deaths per 1000 livebirths.^35^, ^36^

#### 3.2.2 Doubts regarding safety & effectiveness of COVID‐19 vaccine

The general population thinks that the COVID‐19 vaccine is not safe or rather considers the vaccine to be ineffective, despite the sufficient amount of evidence suggesting otherwise. Yasmin et al.'s^37^ study shows that 42% of their surveyed population was concerned about the side effects, while 25% did not think it was effective or would stop the infection.^37^ Al‐Wutayd et al.'s study also indicates this as 72.9% of the vaccine hesitant people said safety was their primary concern.^38^ A cross‐sectional of 423 participants in 2021 suggests that only 37.6% of the vaccine hesitant people thought it was effective (details provided in Table 2).^39^ Furthermore, many women are hesitant to get vaccinated while pregnant due to the perceived implications of the vaccine on the fetus, although studies have shown that the vaccine is safe during pregnancy, and it is the virus that can lead to harmful pregnancy‐related complications.^40^, ^41^ These doubts and health concerns show the need for better communication between the government and the general public.

**Table 2:** Studies highlighting factors influencing COVID‐19 vaccine hesitancy in Pakistan

First author (Year)	Study objective	Study design	Sample size (*n*)	Findings	Limitations
Yasmin et al (2021)	To analyze the COVID‐19 vaccine acceptance rates, beliefs, and barriers impacting vaccination rates among the general population in Pakistan	Cross‐sectional survey	1900	28% people were vaccine hesitant Only 44% people consider the vaccine safe 42% people were vaccine hesitant (VH) due to fear of side effects 25% VH people did not believe the vaccine will stop the infection 44% VH people would get vaccinated if recommended by their physician	Difference in public perceptions that may be associated with sociodemographic factors was not addressed in the study Relative to Pakistan's population sample size is not very large
Al‐Wutayd et al. (2021)	To determine the proportion and predictors of COVID‐19 vaccine hesitancy (VH) among adults in Pakistan	Cross‐sectional study	1014	35.8% people were VH 72.9% VH people were unsure or did not consider the vaccine safe People with household income greater than 2083 USD were 0.59 times less likely to be VH 20.1% people were VH due to conspiracy theories 10% were VH due to fear of needles	The study shows participants intentions to get vaccinated against SARS‐CoV‐2, but public intentions can change over time There may also be social desirability bias in reporting an intent to get vaccinated against SARS‐CoV‐2 The participants were predominantly younger than 40 years old, educated, and employed, and therefore may not be representative of the actual Pakistani population
Chaudhary et al. (2021)	To examine the factors associated with acceptance of the COVID‐19 vaccine compared to hesitance in the Pakistani population and focusing on the perceived beliefs, knowledge, concerns, risk, and safety perception relating to the COVID‐19 vaccine	Cross‐sectional study	423	52% people were accepting of the vaccines 37.6% VH people considered the vaccine effective 62.5% VH people were concerned about the vaccine's safety 8.8% of people who had 5 or less than 5 years of education were vaccine accepting	All the data gathered was from a single institution. This creates inherent bias and limits generalizability of the findings
Malik et al. (2021)	To assess the acceptance of COVID‐19 vaccine in Pakistan among healthcare workers	Cross‐sectional study	5237	5.2% HCWs rejected the vaccine 24.5% HCWs wanted to delay the vaccine until more data was available Female HCWs had 80.7% acceptance rate HCWs from medicine had a 91.9% acceptance rate HCWs with no direct patient contact had a 55.8% refusal rate	The questionnaire was in English, this could have led to selection bias towards English‐literate HCWs The sampling method could have created a selection and social desirability bias among HCWs
Zakar et al. (2022)	To find out the attitude of people towards COVID‐19 vaccination and to assess the factors associated with COVID‐19 vaccine acceptance or hesitancy in the Punjab province	Cross‐sectional survey	1325	From rural areas 14.3% people were vaccinated while 28.9% were not vaccinated Access to four sources of information was 48% among vaccinated people whole 34% among non‐vaccinated people 80.1% people got vaccinated to look after their own health 65.7% people got vaccinated due to health concerns for their loved ones	This survey may have excluded the digitally illiterate people who do not possess a personal cell phone and got their vaccinations registered using someone else's number, thus leading to selection bias Participants could have provided socially desirable answers leading to information bias
Hussain et al. (2021)	To ascertain the acceptance rate and perspective of COVID‐19 vaccination among a representative population in a tertiary care center	Cross‐sectional survey	1500	49% people were accepting of the vaccine 36.9% of the VH people did not believe in COVID‐19 or did not consider it a serious disease 43.1% VH people did not trust the vaccine 7.6% people got vaccinated due to a job requirement	The observations made and data gathered at a specific point was in the background of rapidly changing disease dynamics

#### 3.2.3 Inaccurate beliefs about COVID‐19

The timely development of COVID‐19 vaccines has had a significant impact in reducing the severity of the symptoms and in combating the spread of the virus.^42^ Despite this, two in five Pakistani individuals are reluctant to get the COVID‐19 vaccine.^43^ To begin with, many Pakistanis do not even believe in COVID‐19, and simply regard it as the “flu virus,” and thus do not feel the need to vaccinate against a disease that does not exist.^44^ Additionally, those people who do believe in the virus do not consider it a serious threat.^45^ Misleading figures regarding Pakistan's death rate due to COVID‐19 are to blame for the public's frivolous attitude towards the disease. The death rate was low due to underreporting of cases along with fewer testing, and almost 150 countries conducted more COVID tests per million population than Pakistan.^46^ The country's younger population was another reason for the low death rate. However, many falsely believed that the low death rate was because many Pakistanis had a stronger immune system in general.^46^ Also, around 12.6% of the surveyed population does not consider themselves in danger of contracting COVID‐19 or displaying severe symptoms, especially if they are following precautionary measures.^38^ These inaccurate beliefs about COVID‐19 along with downplaying of the virus' threat by the media has caused many people to question the need for vaccines.

#### 3.2.4 Conspiracy theories regarding COVID‐19 vaccines

Like many other vaccines, COVID‐19 vaccine has inspired its own unique conspiracy theories like “the virus was made in a lab” or “the virus was invented to target Islamic nations so that Jews can rule the world” or “China made the virus to destroy western countries.”^43^ These narratives spread rapidly through social media and many uneducated people fall victim to these. Moreover, the free accessibility of the internet has made it difficult for government officials to combat these rumors, and reports indicate that these rumors are mainly spread by younger, politically extreme, and undereducated people.^43^ Additionally, many vaccines were supplied by China, where the virus originated from. This along with the rapid development of the COVID‐19 vaccine (in 1 year, compared to the 5–10 years average for other vaccines) has further fueled the population's mistrust and skepticism.^47^ This has led to 49% of the population being reluctant to get vaccinated even if it is offered for free.^48^

#### 3.2.5 Vaccine hesitancy among different demographics

Vaccine hesitancy varies greatly among different demographics, as indicated by Al‐Wutayd et al.'s study, which shows that participants who were between ages 31 and 40 years and uneducated were more vaccine hesitant. In contrast, participants coming from higher‐income household were 0.59 times less vaccine hesitant.^38^ Another study, further enforces the fact that higher education and better knowledge about the vaccine lead to greater vaccine acceptance, as 53% of the participants were planning to get vaccinated, most of whom were well educated.^39^

Discrepancies in vaccine hesitancy are also observed between rural and urban population of Pakistan. This is highlighted in Zakar et al.'s^49^ study, where 84.3% of the urban population was vaccinated compared to only 14.3% rural population. Another cross‐sectional study conducted in 2021, further supplements this statement, as acceptance among the surveyed urban population was 50.9% compared to 41% in rural areas.^45^ Further details for both studies are provided in Table 2.

A review by Peyberah E. et al.^50^ highlight COVID‐19 vaccine hesitancy in population with mental health illness, which is otherwise not taken into consideration while discussing about vaccine hesitancy in different types of populations. This review describes how lifestyle modifications due to COVID‐19, such as lockdowns, loss of family members who succumbed to the pandemic and so forth, have affected mental and overall health in general due to weakened immunity secondary to depression, anxiety, and poor sleep quality.^50^ These effects on mental health of the population have influenced the negative behavior towards vaccination leading to a higher rate of hesitancy among mentally ill population. This study also highlighted that addressing these issues led to a rise of vaccine acceptance rate from 58.4% to 93.8%.^50^ Taking this into consideration and the fact that 24 million individuals in Pakistan are in need of mental health assistance it can be deduced that addressing vaccine hesitancy among this group in Pakistan can lead to a better vaccine acceptancy; however, the progress is limited due to lack of research in this field.^51^

#### 3.2.6 Hesitancy among healthcare workers

When the COVID‐19 vaccines first became available, healthcare workers (HCWs) were put on the priority list, considering that they were in close proximity with infected patients and had a significant risk of contracting the disease. A cross‐sectional study of 5237 participants in 2021 shows that 70.2% Pakistani HCWs were positive about getting vaccinated.^52^ Despite the high acceptance rate these figures are worrisome, as medical personnel are highly educated and have higher incomes, therefore, the rates should have been higher. Moreover, any hesitancy displayed by medical staff, who are responsible for recommending and administering the vaccine, would lead to higher public mistrust. This is highlighted in studies by Al‐Wutayd et al. and Yamsin et al., where 13.2% and 44.2% of the vaccine hesitant participants within the study agreed to get vaccinated, respectively, provided that the recommendation was given by a physician.^37^, ^38^

#### 3.2.7 Cost of the vaccine

Apart from the multitude of reasons mentioned above, cost of the vaccine was another factor contributing to hesitancy. Although, many government centers provided free vaccination, cost became an issue. Many did not trust the quality of the free vaccines and opted for the internationally made or imported vaccines, as shown in Yasmin et al.'s study.^37^ This distrust stems from the fact that many Pakistani citizens were unsure why the government would provide free vaccines, when other necessities like clean water, electricity, and gas were still lacking.

COVID‐19 has limited treatment options and the general care provided is oxygen therapy, which is supportive in nature. Hence, there is a critical need to combat vaccine hesitancy swiftly and efficiently, especially in developing countries like Pakistan, where the stagnant economy makes prolonged lockdowns unfeasible for the citizens and the whole nation.^53^

A summary of the various factors discussed above along with the figures and findings from the studies is provided in Table 2.

### 3.3 Other challenges

#### 3.3.1 COVID‐19 testing

In the wake of the global pandemic, the government of Pakistan along with its healthcare authorities has faced many obstacles in reducing the virus' spread. Hesitancy to COVID‐19 testing is among the many reasons. Testing is a key component in understanding the true extent of the pandemic and subsequently coming up with solutions to slow or halt the spread of the disease, as testing can reveal whether flu‐like symptoms are due to influenza or SARS‐CoV‐2.^54^ Although government centers offered free COVID‐19 testing across the country, many people were hesitant to get tested. Fear of needles was also a cause of refusal from 10% of the vaccine hesitant population.^37^ Moreover, many were afraid that they would have to self‐isolate and could miss work, a luxury which many working‐class citizens could not afford.^55^ Additionally, many of the virus' symptoms were general like cough, fever, and headache, which made differential diagnosis a challenge and many assumed they just had the seasonal flu and did not get tested.^56^ On the contrary, those who did get tested had a 38% chance of being false negative.^57^

#### 3.3.2 Public gatherings

Religious occasions have also contributed to the virus' spread. Ten days before Eid in 2020, lockdowns were eased throughout the country so people can meet their families and spend the joyous occasion with their loved ones. However, the decision backfired as number of daily cases in the second week of June, following the religious holiday of Eid, which is celebrated by a majority of the country.^46^, ^58^

#### 3.3.3 Standard operating procedure noncompliance

Noncompliance of standard operating procedures (SOPs) have further increased the virus' rampant spread, as many people do not practice social distancing, wear masks, nor do they wash their hands with proper hand washing techniques. Furthermore, large crowds have been spotted at public gatherings, despite the high transmission rates reported at such overcrowded events.^53^ Compliance rate in industrial sector and mosques was only 38% and 41%, respectively, while in hospitals it was only 70%.^59^ This is alarming as hospitals are where many infected people are quarantined and must have a compliance rate of 100%.^59^

### 3.4 The gray area

Multiple studies showed that many participants feared the vaccines' side effects,^37^, ^38^, ^39^ despite which, very limited data are available on COVID‐19 vaccines' long‐term health implications, which is fair considering the vaccine only became widely available in 2021.^60^ Apart from the vaccine, COVID‐19's long‐term health implications also require more research. This is essential as many of the surveyed participants were also vaccine hesitant because they did not consider COVID‐19 a serious threat to their wellbeing.^38^

### 3.5 Effects on public health

Lower vaccination rates due to COVID‐19 vaccine hesitancy can have several impacts on the public health of Pakistan, as stated by Pakistan's Health Minister, Dr. Sultan. This is because it heightens the risk of resurgences which would devastatingly affect businesses and health across the country. Without the vaccine, we will always be at risk of more outbreaks. That is not to say it cannot happen, but the odds are lower.^61^ Given the devastating impact this pandemic has had on the global economy, if we are to reverse this trend and restore world order, we need to be able to allow the free movement of people, which will only be possible if each individual is immunized to stop the spread of this deadly illness. Emerging evidence also suggests that vaccinated individuals are less likely to contract an asymptomatic infection or transmit SARS‐CoV‐2. This suggests that vaccination will confer some kind of herd immunity.^26^

COVID‐19 has severely disrupted tuberculosis (TB) health services including diagnosis, care, and prevention services. TB service providers across a variety of high TB burden contexts have encountered difficulties in providing services due to lack of available equipment and capacity, restrictions on movement due to the strict lockdowns, as well as the reallocation of resources from TB focused programs to initiatives tackling COVID‐19.^62^ Meanwhile, TB patients have had difficulty receiving treatment due to the fear of SARS‐CoV‐2 infection, stigma, restriction of movement, reduced facility opening hours, or financial constraints.^62^ Studies have further indicated that COVID‐19 coinfection with current or prior TB disease is associated with less favorable COVID‐19 outcomes, such as a two‐ to three‐fold increase in mortality or a 25% relative decrease in recovery.^62^ In terms of COVID‐19 vaccine hesitancy, lower vaccination rates can have a number of detrimental effects on the ongoing TB crisis across the country as COVID spread may mask TB transmission, which renders previous efforts by the country to control TB ineffective. To combat the spread of the disease, many resources have been redirected, which can be used to improve local healthcare facilities, but the low vaccination rates hamper the progress.

Countries seeking to close the immunization gap face an increasing challenge from those who delay or refuse vaccines for themselves or their children. As novel strains of COVID‐19 emerge and impose a threat to everyone's health, vaccine hesitancy and refusal put the lives of others, especially the immunocompromised, at risk.^63^ To reduce their risk of exposure, these people depend upon the general public to be vaccinated.

Not just COVID‐19, but the vaccine may also protect against other immune‐deficiency diseases, such as the common flu and colds with overlapping clinical manifestations including cough, fever, fatigue, runny nose, headache, and sore throat.^64^, ^65^ Receiving the vaccine would not only protect the people against contracting the disease but will also minimize complications in those who do contract it even after vaccination.^64^

Lack of mobility has previously been a major issue among the confined as people kept themselves isolated, ignoring their basic needs.^64^ Social isolation, quarantine, and loneliness have been shown to increase the risk of depression and anxiety, negatively affecting public mental health.^66^ It is therefore crucial that measures should be taken on a national level to circumvent future barriers, and devise effective strategies for vaccinating a sufficient number of individuals around the world to achieve herd immunity and significantly reduce health disparities.

## 4 RECOMMENDATIONS

COVID‐19 vaccine hesitancy is something that should be considered and tackled simultaneously from various and multiple angles. The Pakistani government, hospitals, and educational institutions all have various ways and opportunities to reduce the stigma behind the vaccination and increase vaccination rates across the country. For example, the government has the Pakistan Electronic Media Regulatory Authority, which works together with the Ministry of Information to regulate various media outlets and even has the authority to suspend or cancel these same outlets' licenses.^67^ Therefore, the government has the capabilities to censor and punish any news outlets that purposely spread damaging misinformation surrounding COVID‐19 and its vaccination. This is essential as many of these same news outlets have had conspiracy theorists televised to the general public stating falsehoods such as that the vaccine is a way for Bill Gates and western countries to make Muslims slaves to the United States along with other extremely outlandish claims.^68^ Not only should these news outlets have their license suspended, but those who are responsible for televising such segments should be held accountable and if required, punished, as the misinformation they spread will inevitably lead to fear surrounding the vaccine, thus leading to lower vaccination rates and eventually more cases of COVID‐19 culminating into more COVID‐19‐related deaths. By holding those responsible accountable, it will deter other news outlets from freely spreading false information and will encourage them to put more effort into cross‐checking the very same information before televising it. Furthermore, these same stations should employ educated healthcare professionals to speak on the matter as this engagement between them and the general public will quell any exaggerated statements surrounding the vaccine and clear any doubts or fears of the side effects as these are considerable factors that contribute to the vaccine hesitancy.^69^ These changes would reduce the number of conspiracies and the stigma behind the vaccine as well as reduce the fear behind the side effects of the vaccine, thus leading to a higher vaccination rate, reducing the number of COVID‐19 cases and saving more lives.

Hospitals and educational institutions also have ways they can help improve vaccination rates and reduce vaccination hesitancy among local individuals. According to one study, as discussed previously, only an approximate 70% of HCWs surveyed readily accepted the COVID‐19 vaccination.^52^ Considering one‐third of HCWs do not want to be vaccinated,^37^ it is imperative for hospitals to not only educate their staff but also to then have their staff educate their patients in their institutions on the need and benefits of the vaccine as well as explain any side effects or debunk false narratives surrounding the vaccine. Another such aspect where this is possible is that these HCWs can inform their patients, particular pregnant women, that Society of Obstetricians and Gynecologists of Pakistan recommends the COVID‐19 vaccination for pregnant and breastfeeding women. The same society also found that the vaccine is crucial in preventing a critical COVID‐19 infection which is essential for maintaining the health of both the mother and her unborn child.^40^, ^41^ The educational institutions, especially those that educate in the medical field, can educate their students in the same way. This will in turn lead to the same students bringing the knowledge home and further educating their family members including those who may be skeptical about the vaccine but may trust their relatives who are in such institutions since it comes from a trusted family member. Lastly, although unfortunate, many people do get their information from social media which is a platform rife with misinformation.^70^ Even though it is difficult to limit the spread of this misinformation and to penalize those who do, it is possible to educate the influencers of various social media platforms such that they can share the proper information with their general audience. Even though it is difficult to limit the spread of this misinformation and to penalize those who do, it is possible to educate specific key influencers of various social media platforms such that they can share the proper information with their general audience. It is unreasonable to educate every influencer; however, a task that is more plausible would be to find specific influencers who have a wider audience, are open to discussing vaccinations with their followers, or even influencers who are fostering vaccine hesitancy. By targeting those with a wider audience and/or those who are open to discussing the information and misinformation surrounding the COVID‐19 vaccine a significant number of individuals can be reached and taught accordingly. This can be coupled with reaching out to influencers who have fostered some degree of vaccine hesitancy. While they may be harder to approach, if we can show them the benefits of the COVID‐19 vaccine while dispelling any misconceptions they can spread this newfound knowledge with their followers and bring about the added benefit of possibly encouraging those who are hesitant into those who take up the vaccine. This is essential as research has shown that those who are resistant to taking the COVID‐19 vaccine were less likely to obtain their information from traditional outlets such as news and government agencies due to mistrust and were consequently more likely to get their information from social media.^71^ These actions would circumvent the general public's distrust of the government, their exaggerated fears surrounding the side effects, and the doubts on the efficiency and benefits of the vaccine as well as work to reach the most amount of people as possible.

One aspect of COVID‐19 vaccine hesitancy that's often overlooked is hesitancy in patients with mental illness. There are various mental illnesses and phobias which affect patients in such a way that leads to hesitancy. For example, patients with obsessive‐compulsive disorder will find it difficult to leave their homes, those with paranoia may not trust the vaccine itself, those who administer it, or the government behind it especially with all the misinformation circulating. Additionally, those with delusions may think themselves immune to COVID and other infections. In terms of phobias, those with agoraphobia may find it difficult to travel to vaccination centers and those with trypanophobia who fear needles will be resistant to taking the vaccine altogether. Trypanophobia is particularly of concern as according to a study, 30% of young adults were affected by it.^50^ Therefore it is evident that psychiatrists play an essential role in reducing the vaccine hesitancy in those with mental illnesses and phobias. Psychiatrists are crucial in this sense as they are typically trusted by their patients and having the psychiatrists guide their patients in what impedes them as well as in removing obstacles surrounding the vaccine will help reduce this particular aspect of COVID‐19 hesitancy.

In other countries many private companies offered discounts and promotions to individuals who received the COVID‐19 vaccination.^72^ Similarly, it is possible to encourage local companies to do the same here as many people would follow the trend of availing said promotions and spreading the news to those around them, thus encouraging more people to get vaccinated. This would be beneficial to the same companies as more people being vaccinated would lead to less lockdowns resulting in more consumer traffic with corresponding additional sales. Furthermore, the government themselves can also have their social programs make it mandatory for people to get the vaccine to continue receiving benefits for said programs. This would make it necessary for many people to get the vaccine and reduce vaccine hesitancy.

## 5 LIMITATIONS

Our study had some limitations associated which included selection bias as no systematic review or meta‐analysis was performed, and the studies included were only used to compare and determine the factors associated with COVID‐19 vaccine hesitancy in Pakistan based on their importance. Moreover, like any other narrative review, our study also had a subjective approach with the methodology section and thus the conclusion was also subjective of the authors' interpretation of the sensitivity of the factors.

## 6 CONCLUSION

To conclude, vaccination campaigns have always faced challenges in Pakistan. Persistence of low COVID‐19 vaccination rates warrant the need for timely public health interventions. Several factors influence vaccine hesitancy among Pakistanis, which include misinformation and conspiracies, inaccurate religious beliefs, noncompliance of HCWs which demotivates the population, and uncertainty about the efficacy of the vaccines. Along with the hesitancy, noncompliance of SOPs, and lack of testing further exacerbate the situation. Low COVID‐19 vaccination rates can have several detrimental effects on the public health of Pakistan, such as development of new potentially harmful COVID‐19 strains, mental health deterioration due to lockdowns, and uncontrolled spread of TB. To address these challenges, initiatives include disciplining the media, spreading awareness amongst the youth, elucidate upon the importance of vaccination to the healthcare staff, and promoting social media influencers to spread the accurate knowledge through various platforms as well as provide incentives through the government themselves or via the private sector.

## AUTHOR CONTRIBUTIONS

**Zoaib Habib Tharwani**: Project administration; supervision; writing – original draft. **Prince Kumar**: Writing – original draft. **Wajeeha Bilal Marfani**: Writing – original draft. **Sean Kaisser Shaeen**: Writing – original draft. **Alishba Adnan**: Methodology. **Parvathy Mohanan**: Writing – original draft. **Zarmina Islam**: Writing – review & editing. **Mohammad Yasir Essar**: Conceptualization; supervision; writing – review & editing, All authors have read and approved the final version of the manuscript.

## CONFLICT OF INTEREST

The authors declare no conflict of interest.

## TRANSPARENCY STATEMENT

The lead author Mohammad Yasir Essar affirms that this manuscript is an honest, accurate, and transparent account of the study being reported; that no important aspects of the study have been omitted; and that any discrepancies from the study as planned (and, if relevant, registered) have been explained.

## Data Availability

All data are included in the manuscript, Mohammad Yasir Essar had full access to all of the data in this study and takes complete responsibility for the integrity of the data and the accuracy of the data analysis.”
